# Heart Failure With Preserved Ejection Fraction: Current Status of Daily Clinical Practice in Indonesia

**DOI:** 10.7759/cureus.38086

**Published:** 2023-04-24

**Authors:** Siti E Nauli, Vebiona K Prima Putri, Habibie Arifianto, Hawani S Prameswari, Anggia C Lubis, Edrian Zulkarnain, Dian Y Hasanah, Paskariatne P Dewi Yamin, Triwedya I Dewi, Irnizarifka .

**Affiliations:** 1 Department of Cardiology, Tangerang District Hospital, Tangerang, IDN; 2 Working Group on Heart Failure and Cardiometabolic Disease, Indonesian Heart Association, West Jakarta, IDN; 3 Department of Cardiology, Awal Bros Hospital, Pekanbaru, IDN; 4 Department of Cardiology, Universitas Sebelas Maret Hospital, Surakarta, IDN; 5 Department of Cardiology, Hasan Sadikin General Hospital, Bandung, IDN; 6 Department of Cardiology, Haji Adam Malik General Hospital, Medan, IDN; 7 Department of Cardiology, Mohammad Hoesin General Hospital, Palembang, IDN; 8 Department of Cardiology, National Cardiovascular Center Harapan Kita, West Jakarta, IDN; 9 Department of Cardiology, Gatot Soebroto Central Army Hospital, Central Jakarta, IDN; 10 Department of Cardiology, Universitas Sebelas Maret Hospital, Sukoharjo, IDN

**Keywords:** perception, physician knowledge, preserved ejection fraction, ejection fraction, heart failure

## Abstract

Introduction

Heart failure (HF) is a clinical syndrome with symptoms and/or signs caused by a structural and/or functional cardiac abnormality and corroborated by elevated natriuretic peptide levels and/or objective evidence of pulmonary or systemic congestion. Among HF types, HF with preserved ejection fraction (HFpEF) is the commonest form. However, the diagnosis and management of HFpEF are challenging. In addition, the perception of healthcare professionals (HCPs) towards the diagnosis and management of HFpEF patients differs due to the existing gap between the guidelines and daily clinical practice. Therefore, an online survey was conducted to understand the HCPs’ knowledge and practice gaps in the diagnosis, treatment, and management of patients with HFpEF.

Methods

A total of 160 respondents, i.e., cardiologists, internists, and cardiology residents from different community-based practices and hospitals across Indonesia participated in an online continuing medical education (CME) survey. A questionnaire was formulated to assess awareness, current practice patterns, challenges, and confidence of the HCPs related to the HFpEF.

Results

HCPs stated that diagnosis of HF is the prime responsibility of cardiologists and general physicians but not of general internists. According to the HCPs, reduction in mortality, reduction in hospitalization, and improved quality of life are the most important goals of HF treatment. The perceived prevalence of HFpEF is estimated to be 30-60% and mortality rates of HFpEF and HF with reduced ejection fraction (HFrEF) are similar. Further, mixed types of responses with different combinations of diagnosis, treatment, and prevention, were obtained when HCPs were asked about the challenges faced in HFpEF. Among the therapies, angiotensin-converting enzyme (ACE) inhibitors, mineralocorticoid receptor antagonists (MRA), beta-blockers, and diuretics are frequently used for the treatment of HF.

Conclusion

The perception of the HCPs toward the diagnosis and management of HFpEF may affect optimal care. Based on our findings, the cardiologists are well aware of the current situation of HF in Indonesia and treat patients with HFpEF effectively.

## Introduction

Heart failure (HF) is a complex syndrome associated with increased mortality and morbidity. It is characterized by decreased functionality of heart muscles to pump blood, resulting in reduced quality of life. It has affected approximately 38 million people worldwide and 10 million people in Indonesia [1,2]. Studies in the literature reported the estimated prevalence of HF in Indonesia is around 5% [2]. It is responsible for the hospitalization of approximately 1.8 million people per year. The 30-day mortality, hospital mortality, and re-admission rates in Indonesia were estimated to be 17%, 3%, and 29%, respectively, in patients with chronic HF [1,3]. The financial burden of HF is high due to increased hospitalization and readmission rates and is expected to be increasing substantially worldwide.

In 2020, the members of the Heart Failure Society of America (HFSA), the Heart Failure Association of the European Society of Cardiology (HFA/ESC), and the Japanese Heart Failure Society (JHFS) consensually defined HF as “a clinical syndrome with symptoms and/or signs caused by a structural and/or functional cardiac abnormality and corroborated by elevated natriuretic peptide levels and/or objective evidence of pulmonary or systemic congestion” [4]. Based on the calculated left ventricular ejection fraction (EF), HF is classified as (1) HF with reduced EF (HFrEF; EF ≤40%), (2) HF with preserved EF (HFpEF; EF ≥50%), (3) HF with mid-range EF (HFmrEF; EF 41-49%), and (4) HF with improved EF (HFimpEF; baseline EF ≤40% and a second EF >40%) [4]. A report from the Sardjito Heart Failure Registry stated that the prevalence of HFpEF in Indonesia was 43%. In general, the ejection fraction values are considered the strongest prognostic factors, however, diagnosing patients with HFpEF is more challenging clinically [5,6].

For effective diagnosis of the disease and management of patients with HF or particularly HFpEF, several guidelines, such as ESC and American Heart Association (AHA)/American College of Cardiology (ACC), have recommended various diagnostic tools to determine prognosis or disease severity and evidence-based approach, respectively. For instance, left ventricle (LV) structural or functional alterations, such as elevated natriuretic peptide levels, filling pressures, or hemodynamic measurements, may support the diagnosis of HFpEF. Further, the use of diuretics, sodium-glucose cotransporter 2 inhibitor (SGLT2i), angiotensin receptor-neprilysin inhibitor (ARNi), mineralocorticoid receptor antagonist (MRA), and angiotensin receptor blocker (ARB) are recommended therapies for the management of patients with HFpEF [7]. However, HFpEF diagnosis and management in developing countries like Indonesia is difficult due to nonspecific signs and symptoms (such as fatigue, breathlessness, and swelling in the ankle, etc.), diagnostic uncertainty (measures such as mitral annular early diastolic velocity {e′}, LV mass index, relative wall thickness, LV filling pressure estimated using E/e′, and serum natriuretic peptide levels, etc.), existing co-morbidities, and lack of resources to perform additional examinations. Notably, the perception of healthcare professionals (HCPs) towards the diagnosis and management of HFpEF patients also differs due to the existing gap between the guidelines and daily clinical practice. Till date, Indonesian data on HFpEF phenotype is also very limited.

Based on this background, the present study aimed to understand the perceptions of HCPs in terms of awareness, current practice patterns, challenges, and confidence related to the diagnosis, treatment, and management of patients with HFpEF, via an online survey to decipher the HCPs knowledge and practice gaps.

## Materials and methods

An online continuing medical education (CME) survey was conducted to assess awareness, current practice patterns, challenges, and confidence of HCPs related to the diagnosis, treatment, and management of patients with HFpEF.

Survey designing and development

Based on the pertinent literature, ESC (2021) and ACC/AHA (2020) guidelines, and expertise in HF management, a questionnaire was formulated by a team of experts from the Indonesian Heart Failure Working Group. The Indonesian Heart Failure Working Group works under the Indonesian Heart Association (IHA) which aims to lower the National burden of heart failure and cardiometabolic disease [8]. The survey was based on the current challenges, burden, new treatments, guidelines, potential knowledge/awareness, goals, and practice gaps. The questionnaire comprised of 17 items including multiple choice, yes or no, and free text questions related to HF and HFpEF specific. In addition, a case-based competence question was also included to assess the alignment of diagnosis with evidence. The included survey questions were approved by all members of the expert team. The final survey was also reviewed and approved by additional experts. The survey was approximately 10 minutes long and was conducted between March 18, 2022, and June 24, 2022. The questionnaire was distributed electronically to HCPs across Indonesia. The demographic data including age, gender, practice location, and specialty of the HCPs were also recorded.

Respondents

A total of 160 respondents, i.e., cardiologists, internists, and cardiology residents from different community-based practices and hospitals across Indonesia participated in the survey. The respondent’s identity, practice location, and responses were kept anonymous and confidential. Additionally, the respondents were informed that answering the questionnaire would mean their consent to participate in the survey.

Statistical analyses

Only completed surveys were included for statistical analysis. All data were analyzed using GraphPad version 8 (San Diago, CA: GraphPad Software Inc.). The Kolmogorov-Smirnov Goodness of Fit test was used to assess the normality of the data. Based on the data, the results are reported in frequencies and percentages. The data generated by multiple choice questions were analyzed using descriptive statistics.

## Results

Demographic characteristics

In total, 160 participants completed the survey. Among them, 87.5% (n=140) were cardiologists, 11.25% (n=18) were cardiology residents and two respondents were identified as internists. Of them, 55% (n=88) belonged to the age group of <40 years, 25% (n=40) were in the age range of 40-49 years, and the remaining 20% (n=32) respondents were 50 years or above. About 63.1% (n=101) of respondents were males and 36.9% (n=59) were females. Although HCPs from all the regions of Indonesia participated and most respondents were from Jakarta, Java, Bali, Sumatra, Banten, and Sulawesi. Notably, HCPs had been practicing for more than 10 years and treating >10 patients with HF per week. The demographic data of the respondents are detailed in Table 1.

**Table 1:** Demographic data of the study subjects. n: number of subjects

Characteristics, n (%)		Respondents (n=160)
Specialty	Cardiologists	140 (87.5)
	Cardiology residents	18 (11.25)
	Internists	02 (1.25)
Age group	<40	88 (55)
	40-49	40 (25)
	>50	32 (20)
Gender	Male	101 (63.13)
	Female	59 (36.87)
Practice’s location	Java Island	
	Jakarta	33 (20.6)
	Java	43 (26.8)
	Banten	08 (5)
	Yogyakarta SR	05 (3.12)
	Sumatra Island	
	Lampung	02 (1.25)
	Aceh	03 (1.87)
	Sumatra	10 (6.25)
	Riau	04 (2.5)
	Bengkulu	01 (0.62)
	Jambi	02 (1.25)
	Others	
	Borneo	05 (3.12)
	Bali	34 (21.25)
	Sulawesi	08 (5)
	Maluku	01 (0.62)
	Nusa Tenggara	01 (0.62)

Heart failure diagnosis and management

**Figure 1:**
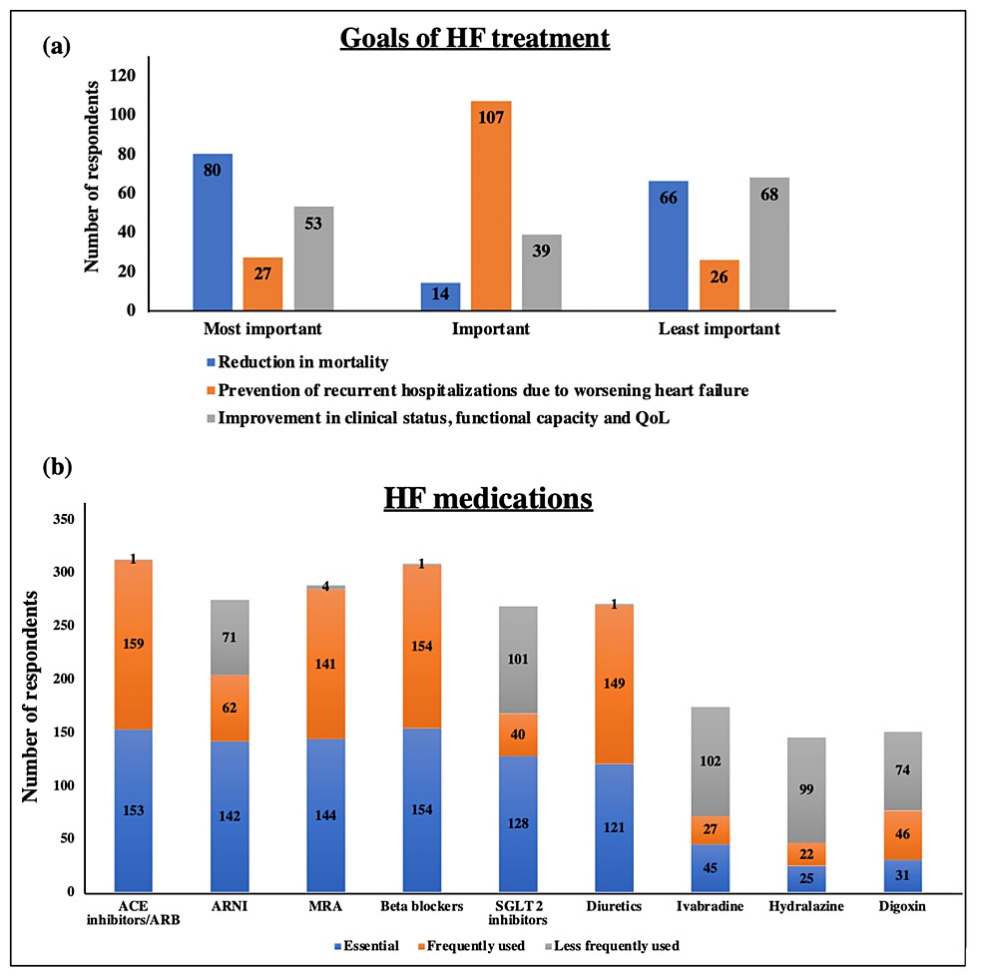
Goals and medications used in the management of HF. (a) Bar-graph represents the frequency of respondent ratings on the goals of HF treatment in terms of reduction in mortality, prevention of recurrent hospitalizations due to worsening heart failure, and improvement in clinical status, functional capacity, and quality of life. (b) Bar-graph represents the frequency of respondents stating essential, frequently used, and less frequently used medications. HF: heart failure; ACE: angiotensin-converting enzyme; ARNi: angiotensin receptor-neprilysin inhibitor; MRA: mineralocorticoid receptor antagonist; SGLT2: sodium-glucose cotransporter 2; QoL: quality of life

Based on the responses, 54.4% and 45.6% of respondents stated that the diagnosis of HF is the prime responsibility of cardiologists and general physicians, respectively. However, none of the respondents stated it as the responsibility of the general internist. When the respondents were asked to rank the goals of HF treatment, approximately 50% of respondents rated reduction in mortality as the most important goal followed by prevention of recurrent hospitalizations due to worsening heart failure, and then improvement in clinical status, functional capacity, and quality of life (QoL) (Figure 1, panel a).

Further, a list of HF medications, such as angiotensin-converting enzyme (ACE) inhibitors, ARB, beta-blockers, MRA, ivabradine, hydralazine/isosorbide dinitrate, SGLT2 inhibitors, ARNi, diuretics, and digoxin was given and asked to mark the essential, frequently used, and less frequently used medications. More than 75% of respondents stated that ACE inhibitors, ARNI, MRA, beta-blockers, SGLT2 inhibitors, and diuretics are essential medications for HF management. Among them, ACE inhibitors, MRA, beta-blockers, and diuretics are frequently used HF medications, as stated by >85% of respondents. Based on their responses, ivabradine, hydralazine/isosorbide dinitrate, SGLT2 inhibitors, ARNI, and digoxin are less frequently used HF medications. The data is shown in Figure 1, panel b.

HFpEF management

**Figure 2:**
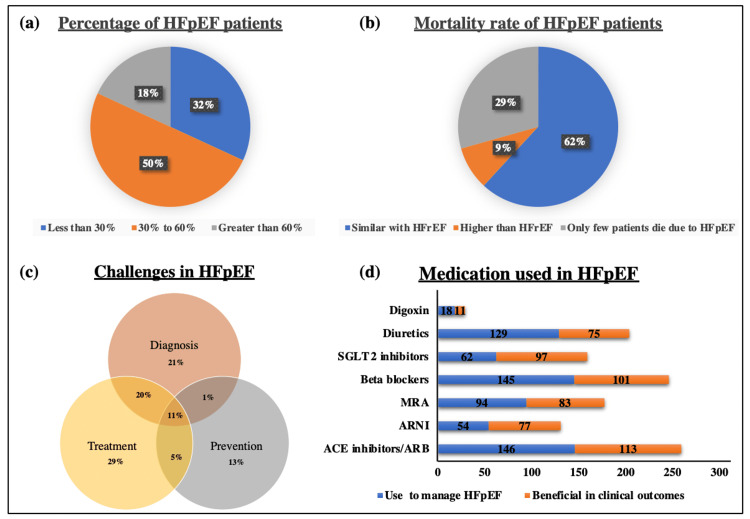
Management of HFpEF. (a) Pie-chart displays the percentage of respondents seeing HFpEF patients among all HF patients. (b) Pie-chart displays the percentage of respondents’ awareness of the mortality rate of HFpEF. (c) Venn diagram delineates the percentage of respondents facing challenges in the management of HFpEF. (d) Sidebar diagram represents the frequency of respondents using medications to manage HFpEF and which are beneficial in better clinical outcomes of HFpEF. HFpEF: heart failure with preserved ejection fraction; ACE: angiotensin-converting enzyme; ARNi: angiotensin receptor-neprilysin inhibitor; MRA: mineralocorticoid receptor antagonist; SGLT2: sodium-glucose cotransporter 2; ARB: angiotensin receptor blocker; HFrEF: heart failure with reduced ejection fraction

When HCPs were asked about the percentage of patients with HFpEF among their HF patients, 80/160 (50%) HCPs reported that 30-60% of patients were with HFpEF, 51/160 (32%) HCPs reported the HFpEF patient percentage less than 30%, while the remaining 18% stated the percentage of greater than 60%. Regarding the awareness of HCPs on the mortality rate of HFpEF patients, 62% of respondents stated that the mortality rate among HFpEF patients is similar to HFrEF while 30% of respondents stated that only a few patients die due to HFpEF (Figure 2, panel a, b).

When asked about the challenges faced in HFpEF, 30% of respondents indicated “treatment” as a major challenge followed by “diagnosis” and then “prevention.” About 37% of respondents stated combination of diagnosis and treatment, prevention and treatment, and diagnosis, prevention, and treatment as challenges in the management of HFpEF. The responses of the participants are shown in the form of a Venn diagram (Figure 2, panel c). Further, about 79% of specialists reported that they find more difficulty in treating patients with HFpEF than patients with HFrEF while the remaining 21% of specialists did not find any difficulty.

**Figure 3:**
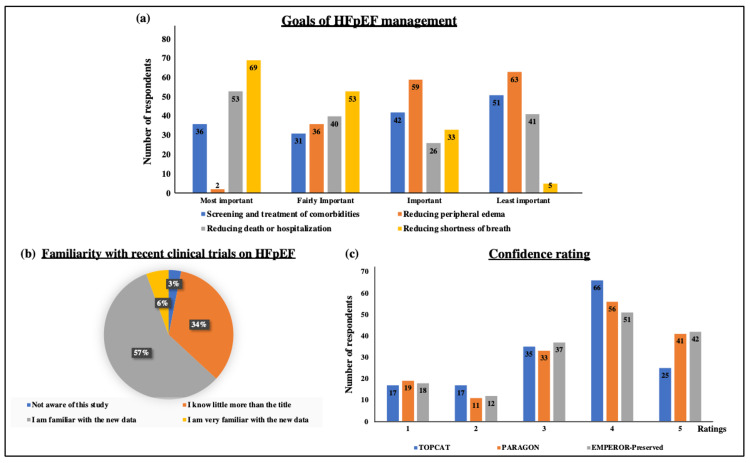
Goals of HFpEF management and awareness related to recent clinical trials. (a) Bar graph represents the frequency of respondent ratings on the goals of HFpEF management in terms of screening and treatment of comorbidities, reducing death or hospitalization, reducing shortness of breath, and reducing peripheral edema. (b) Pie chart represents the percentage of respondents stating familiarity with recent clinical trials on HFpEF. (c) Bar graph represents the frequency of respondent ratings on their confidence in prescribing medications based on the outcomes of clinical trials. HFpEF: heart failure with preserved ejection fraction

When the list of medications was given to the physician for the management of HFpEF, more than 80% of physicians named ACE inhibitors, beta-blockers, and diuretics as major medicines used and proven to be beneficial on the clinical outcomes in HFpEF (Figure 2, panel d). Further, the respondents were asked to rank the goals in managing HFpEF in terms of screening and treatment of comorbidities, reducing death or hospitalization, reducing shortness of breath, and reducing peripheral edema. Mixed responses were obtained which are shown in Figures 3a-3c.

Based on recent clinical trials of HFpEF such as TOPCAT [9], PARAGON [10], and EMPEROR-Preserved [11], the respondents were asked if they are familiar with the results of these clinical trials and if they are confident to give those treatments to the patients. About 57% of the respondents stated that they are familiar with the new data and 70-80% of physicians are fairly to extremely confident to give those treatments (Figures 3a-3c). As an open question, respondents are willing to learn more about diagnosis criteria, new treatment strategies, clinical trials, updated guidelines, and new biomarkers.

Case analysis

**Figure 4:**
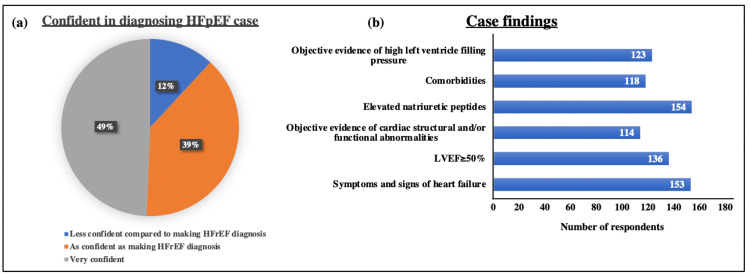
Case analysis. (a) Pie chart displays the percentage of respondents who were confident in diagnosing HFpEF in the given case. (b) Sidebar diagram represents the number of respondents marking the findings assessed from the case in terms of symptoms and signs of heart failure, LVEF ≥50%, objective evidence of cardiac structural and/or functional abnormalities, elevated natriuretic peptides, comorbidities, and objective evidence of high left ventricle filling pressure. HFpEF: heart failure with preserved ejection fraction; LVEF: left ventricular ejection fraction

In the survey, a case was presented as an 85-year-old female with leg edema and paroxysmal atrial fibrillation. She had a history of hypertension and the clinical reports showed EF as 56%, E/e’ as 16, and NT-proBNP as 2700 pg/mL. The respondents were asked about their confidence in diagnosing HFpEF in this case and the associated findings. Approximately 88% of respondents stated that they are very confident or as confident as making an HFrEF diagnosis while 12% of respondents were less confident. Further, 70% and above respondents stated all the findings assessed from this patient, i.e., symptoms and signs of heart failure, left ventricular ejection fraction (LVEF) ≥50%, objective evidence of cardiac structural and/or functional abnormalities, elevated natriuretic peptides, comorbidities, and objective evidence of high left ventricle filling pressure (Figures 4a, 4b).

## Discussion

HF is a leading medical condition with substantially increasing prevalence in both developing and developed countries [12]. Among the HF types, patients with HFpEF alone account for 50% of affected patients [13]. Despite being the commonest form, HFpEF is a heterogenous condition that challenges cardiovascular medicine in everyday practice [14]. The major goals of HFpEF diagnosis and treatment are reduced exacerbations, hospitalization, re-admissions, and mortality rates to improve functional capacity and quality of life [15]. However, the diagnosis and management of HF are quite challenging due to existing gaps in the guidelines and daily clinical practice. Thus, physicians must be competent to diagnose and manage this complicated disease.

To the best of our knowledge, this is the first Indonesian survey that assessed the HCPs’ knowledge and awareness concerning diagnosis, treatment, and management of patients with HF focusing HFpEF. In the present study, 99% (n=158) of the participants belonged to the cardiology department with a male predominance of 63%. More than half of the HCPs (55%) were <40 years of age. All the major regions of Indonesia were covered during the survey and the majority of the respondents were from Jakarta, Java, Bali, Sumatra, Banten, and Sulawesi. A similar study was conducted by Gupta et al. with 448 physicians from different regions of Canada [16].

Regarding the diagnosis of HF, the participants responded that it is the prime responsibility of cardiologists and general physicians but not general internists. In a study, Hancock et al. conducted a national survey in England to assess changes in healthcare professionals’ views about the diagnosis and management of HF since 2003 [17]. The authors stated that the responsibility of diagnosing HF is of cardiologists followed by general physicians. In another favored study, Smeets et al. carried out an exploratory qualitative study to assess the perception of general practitioners (GP) about their roles in HF care [18]. The authors revealed that GP saw cardiologists as professionals to diagnose and manage patients with HF. The findings are in concordance with our study.

Further, the respondents were asked to rank the goals of HF treatment, approximately 50% of respondents rated reduction in mortality as the most important goal followed by prevention of recurrent hospitalizations due to worsening heart failure, and then improvement in clinical status, functional capacity, and quality of life (QoL) (Figure 1, panel a). The order of the goals varied in the remaining cases. In an editorial comment by Okunade, the author stated that mortality reduction, reduced cardiovascular events, and improve health-related QoL outcomes are the prioritizing goals of HF therapy, which are similar to our results [19].

Concerning HFpEF, the knowledge and awareness of the HCPs were checked and they asked about the prevalence and mortality rate of HFpEF. About 50% of HCPs reported the prevalence of HFpEF at their clinic is between 30% and 60% which is in accordance with the data of Tromp et al., Mumpuni et al., and ESC guidelines 2022 [5,6]. These studies suggest that HFpEF accounts for at least 50% of the HF population, and its prevalence is increasing. Most of the physicians were correctly aware of the mortality rate, i.e., mortality rate among HFpEF patients is similar to HFrEF. In the study of Gupta et al., the majority of the physicians believed that mortality rates were similar between HFrEF and HFpEF [11]. Evidence in the literature also suggests that both HFrEF and HFpEF have comparable mortality rates ranging between 20% and 25%, which is in accordance with our data [16,20]. Further, mixed types of responses with different combinations of diagnosis, treatment, and prevention were obtained when physicians were asked about the challenges faced in HFpEF.

Then, physicians were asked about the difficulty in treating patients with HFpEF. Majority (79%) of the physicians agreed to find more difficulty in treating patients with HFpEF than patients with HFrEF. This is because patients with HFrEF can be readily diagnosed and the condition can be confirmed through cardiac imaging [4]. However, the diagnosis of HFpEF is difficult clinically.

In a list of HF medications, the respondents were asked to mark the essential, frequently used, and less frequently used medications among ACE inhibitors (ACEi), beta-blockers, MRA, ivabradine, hydralazine/isosorbide dinitrate, SGLT2 inhibitors, ARNi, diuretics, and digoxin. Mixed responses were obtained. More than 75% of respondents marked ACEi, ARNi, MRA, beta-blockers, SGLT2 inhibitors, and diuretics as essential medications. Among these, ACE inhibitors, MRA, beta-blockers, and diuretics are frequently used and ivabradine, hydralazine/isosorbide dinitrate, SGLT2 inhibitors, ARNi, and digoxin are less frequently used HF medications. In a study, Reyes et al. reported that ACE inhibitors and diuretics are the major pharmacological treatments for HF in Indonesia [1]. When the list of medications was given to HCPs for the management of HFpEF, more than 80% of HCPs named ACE inhibitors, beta-blockers, and diuretics as major medicines used and proven to be beneficial for the clinical outcomes in HFpEF. Similar responses from the physicians were obtained in the study by Gupta et al. [16]. The AHA guidelines also recommended these medications for the treatment of HF [7]. However, there are no evidence-based treatments available for patients with HFpEF to improve cardiovascular outcomes.

Our study has a few following limitations: (1) the sample size is very small for a large country like Indonesia. (2) Only two internists participated in the survey which limited the availability of comparative data of cardiologists and internists. (3) Mixed responses were obtained for many questions which could not be interpreted or discussed.

## Conclusions

Conclusively, several guideline-endorsed therapies exist to improve the condition of patients with HF. These guidelines are regularly updated and recommend differential diagnosing and treatment strategies with dissemination programs for physicians. The perception of the physician toward the diagnosis and management of HFpEF may affect optimal care. Narrowing the existing gap between knowledge and practice might support new initiatives in the optimization of diagnosing, treatment, and management strategies in patients with HFpEF.

## Appendices

General heart failure questions

A. Awareness of Heart Failure Burden

1. Rank the following goals of heart failure treatment in order of importance (1 = most important, 3 = least important):

__ Reduction in mortality __ Prevention of recurrent hospitalizations due to worsening heart failure __ Improvement in clinical status, functional capacity, and quality of life (QoL).

2. Diagnosis of heart failure is the primary responsibility of (multiple answers):

a. Cardiologist

b. General physician

c. General internist

B. Awareness of Heart Failure (HF) Medication

3. Which heart failure medications are essential (check all that apply):

__ ACE inhibitors/ARB __ ARNi __ MRA __ Beta-blockers __ SGLT2 inhibitors __ Diuretics __ Ivabradine __ Hydralazine/isosorbide dinitrate __ Digoxin

4. Which heart failure medications do you use frequently (check all that apply):

__ ACE inhibitors/ARB __ ARNi __ MRA __ Beta-blockers __ SGLT2 inhibitors __ Diuretics __ Ivabradine __ Hydralazine/isosorbide dinitrate __ Digoxin

5. Which heart failure medications do you use less frequently (check all that apply):

__ ACE inhibitors/ARB __ ARNi __ MRA __ Beta-blockers __ SGLT2 inhibitors __ Diuretics __ Ivabradine __ Hydralazine/isosorbide dinitrate __ Digoxin

HFpEF-specific questions

*A. Awareness of HFpEF Burden and Challenges* 

1. Among your heart failure patients, what is the percentage of patients with HFpEF?

a. Less than 30%

b. 30% to 60%

c. Greater than 60%

2. Are you aware of mortality rate among HFpEF patients?

a. Similar to HFrEF

b. Higher than HFrEF

c. Only a few patients die due to HFpEF

3. What are the challenges in HFpEF based on your opinion?

___ Diagnosis

___ Prevention

___ Treatment

B. Case Analysis - Alignment Diagnosis to Evidence

Case: Female, 85 years old, with leg edema, paroxysmal atrial fibrillation, history of hypertension, EF showed 56%, E/e’ 16, NT-proBNP 2700 pg/mL.

4. How confident are you in diagnosing HFpEF in this case?

a. Less confident compared to making HFrEF diagnosis

b. As confident as making HFrEF diagnosis

c. Very confident

5. What findings you can assess from this patient? (check all that apply):

__ Symptoms and signs of heart failure __ LVEF ≥50% __ Objective evidence of cardiac structural and/or functional abnormalities __ Elevated natriuretic peptides

__ Comorbidities

__ Objective evidence of high left ventricle filling pressure

C. Subjective on Difficulty in Diagnosis HFpEF

6. Are you finding it more difficult to adequately treat patients with HpEF than patients with HFrEF?

a. Yes

b. No

D. Goal of HFpEF Treatment

7. In order of priority, rank the following goals in managing HFpEF (1 = most important, 4 = least important)

__ Reducing shortness of breath __ Reducing peripheral edema __ Screening and treatment of comorbidities __ Reducing death or hospitalization

E. Awareness of HFpEF Medication

8. Which medications do you use in managing HFpEF (check all that apply)?

__ ACE inhibitors/ARB __ ARNi __ MRA __ Beta-blockers __ SGLT2 inhibitors __ Diuretics __ Digoxin

9. To your knowledge, which of the following medications have proven benefits on clinical outcomes in HFpEF (check all that apply)?

__ ACE inhibitors/ARB __ ARNi __ MRA __ Beta-blockers __ SGLT2 inhibitors __ Diuretics __ Digoxin

F. Awareness of Latest Update HFpEF Treatment

10. According to recent clinical trials of HFpEF treatment (TOPCAT, PARAGON, EMPEROR-Preserved) how familiar are you with those results?

a. Not aware of this study

b. I know little more than the title

c. I am familiar with the new data

d. I am very familiar with the new data

11. How confident are you in giving those treatments based on their clinical trial results? (Please use rating scale from 1-5, where 1 = confident and 5 = extremely confident)

a. TOPCAT (spironolactone) ____

b. PARAGON (sacubitril/valsartan) ____

c. EMPEROR-Preserved (empagliflozin) ____

12. If you need information HFpEF, what kind of knowledge that you are willing to learn?
